# The Pronounced Th17 Profile in Systemic Sclerosis (SSc) Together with Intracellular Expression of TGFβ and IFNγ Distinguishes SSc Phenotypes

**DOI:** 10.1371/journal.pone.0005903

**Published:** 2009-06-17

**Authors:** Timothy R. D. J. Radstake, Lenny van Bon, Jasper Broen, Anila Hussiani, Roger Hesselstrand, Dirk M. Wuttge, Yanhui Deng, Robbert Simms, Erik Lubberts, Robert Lafyatis

**Affiliations:** 1 Department of Rheumatology, Radboud University Nijmegen Medical Center, Nijmegen, The Netherlands; 2 The Arthritis Center, Boston University School of Medicine, Boston, Massachusetts, United States of America; 3 BUMC Flow Cytometry Core Facility, Boston University School of Medicine, Boston, Massachusetts, United States of America; 4 Department of Rheumatology, Lund University Hospital, Lund, Sweden; 5 Erasmus MC, Departments of Immunology and Rheumatology, University Medical Center Rotterdam, Rotterdam, The Netherlands; New York University School of Medicine, United States of America

## Abstract

**Background:**

Systemic sclerosis (SSc) is an autoimmune disease where controversy on Th1/Th2 balance dominates. We investigated whether the recently discovered Th17 pattern was present in SSc.

**Methodology and Principal Findings:**

Patients were subdivided as having limited cutaneous SSc (lcSSc, n = 12) or diffuse cutaneous SSc (dcSSc, n = 24). A further arbitrary subdivision was made between early dcSSc (n = 11) and late dcSSc (n = 13) based upon the duration of disease. As a comparator group 14 healthy controls were studied. CD3+ cells were isolated using FACS and subsequently studied for the expression of CD4, CD8, CD25, CD45Ro, CD45Ra, IL-23, GITR, CD69 and intracellular expression of IL-17, TGFβ and IFNγ using flow cytometry. Levels of IL-17, IL-6, IL-1α and IL-23 were measured using Bioplex assays. SSc patients had more and more activated CD4+ cells. In addition, CD4, CD45Ro and CD45Ra cells from all SSc patients highly expressed the IL23R, which was associated with a higher IL-17 expression as well. In contrast, IFNγ and TGFβ were selectively up regulated in SSc subsets. In line with these observation, circulating levels of IL-17 inducing cytokines IL-6, IL-23 and IL-1α were increased in all or subsets of SSc patients.

**Conclusion and Significance:**

The combination of IL-17, IFNγ and TGFβ levels in CD45Ro and CD45Ra cells from SSc patients is useful to distinguish between lSSc, ldSSc or edSSc. Blocking Th17 inducing cytokines such as IL-6 and IL-23 may provide a useful tool to intervene in the progression of SSc.

## Introduction

Systemic Sclerosis (SSc) is a complex inflammatory autoimmune disease characterized by excessive deposition of matrix molecules, leading to fibrosis of multiple organs including the skin, lungs, heart and gastrointestinal tract, and often leading to severe morbidity and premature death. Although the role of immune dysfunction in the pathogenesis of SSc is generally accepted, the exact pathways that cause immune dysfunction in SSc remain to be elucidated. Alterations in cellular immunity are typified by aberrant T cell biology both in the skin as well as circulation of SSc patients. For example, CD4+ T cells are increased in the circulation of SSc patients [1], [2] whereas NKT cells and γ/δ T cells are decreased [3]. In addition, lesional skin from SSc patients displays various features consistent with T cell activation [1], [4], [5]. Finally, T cell biology was altered in SSc in that the secretion of various inflammatory mediators is markedly increased [6], [7].

In this line the Th1/Th2 paradigm has been investigated by studying the presence of Th1 (IL-12, IFNγ) and Th2 (IL-4, IL-13 and IL-10) associated cytokines in the circulation, in circulating cells and in the skin of SSc patients. Driven by opposing findings, these studies led controversy whether these Th1/Th2 profiles could explain the pathogenesis of SSc. The recognition of IL-17 producing T cells (Th17) has opened novel pathways to explain several features of SSc. In general, T cell priming by professional antigen presenting cells is tuned by inflammatory mediators, including TGFβ, IL-6 and IL-12. The combination of these cytokines determines the ultimate fate of naive T cells. For instance, TGFβ alone up regulates FoxP3 expression, a marker for T regulatory cells. In contrast, accumulating evidence suggests that TGFβ in combination with IL-1α, IL-6 or IL-23 drives the expression of RORγt, a proliferation factor specific for the recently identified Th17 subset [8], [9], [10], [11]. Intriguingly, IL-23, IL-1α and IL-17 have been found increased in the circulation of SSc patients compared to healthy controls [12], [13], [14], [15]. Together, these observations suggest the potential for skewing of the Th17 axis in SSc.

Th17 cells are characterized by the production of IL-17A (IL-17) and are thought to clear extracellular pathogens not effectively cleared by either Th1 or Th2 cells. To this aim, Th17 cells appear at sites of inflammation with rapid kinetics and possibly bridge the gap between innate and adaptive immunity by attracting other Th cells to the inflammatory site. Various recent studies have emerged suggesting that Th17 cells are essential in autoimmune diseases. First, mice deficient for the Th1 effector cytokine IFNγ develop enhanced experimental autoimmune encephalomyelitis (EAE) [16], and the absence of IL-23, results in a lack of Th17 cells and protection from EAE and collagen-induced arthritis (CIA) [17], [18]. Second, IL-17 has been found to be increased in patients with rheumatoid arthritis [19], multiple sclerosis [20], inflammatory bowel disease [21], psoriasis [11] and seronegative spondylarthritides [22]. IL-17 has been involved in many pathological features that play a role in SSc pathology including the secretion of pro-inflammatory cytokines, the recruitment of monocytes and the triggering of granulocyte-macrophage colony-stimulating factor [23], [24]. In light of fibrosis being the cardinal feature of SSc, it is interesting to note that IL-17 has also been implicated in fibrosis of the basal membrane in asthma [25] and the control of inflammatory response after bleomycin-induce lung injury, a model often exploited to study pulmonary fibrosis [26].

To address the possible role of IL17 in SSc, we investigated Th17 cell frequency in the circulation of SSc patients, and the expression of key cytokine regulators and markers of T cell phenotypes, IFNγ and TGFβ. Because there are two clinically distinct forms of systemic sclerosis and at least one of these forms evolves over time, we evaluated Th17 cell frequency and cytokine expression in three subgroups: patients with early compared to late diffuse cutaneous SSc and patients with limited cutaneous SSc. We found that circulating Th17 cells are significantly increased in all three SSc patient subsets compared to healthy controls. In addition, together with the expression of IL-17, clinical SSc phenotypes were associated with specific patterns of intracellular expression of TGFβ and IFNγ. Together these data indicate that T cell priming in SSc is skewed towards the Th17 axis, which together with intracellular staining for TGFβ and IFNγ provide a novel markers of SSc phenotypes. Importantly, circulating levels of IL-17 were undetectable whereas the Th17 inducing cytokines IL-6 and IL-23 levels were increased in the circulation of SSc patients.

## Methods

### Study population

#### Cell-based *In vitro* experiments

Thirty-six patients presenting to the Arthritis Center, Boston Medical Center were included in the study **(** **Table 1** **)**. All of the patients met the American College of Rheumatology preliminary criteria for the classification of SSc [27]. Patients were subdivided as having limited cutaneous SSc (lcSSc, n = 12) or diffuse cutaneous SSc (dcSSc, n = 24) on the basis of the extent of their skin involvement [28]. A further subdivision was made between early dcSSc (n = 11) and late dcSSc (n = 13) based upon the duration of disease, defining early dcSSc as patients having a disease duration <2 years and late dcSSc as patients having a disease duration longer than 3 years. As a comparator group 14 healthy controls were studied. Treatment was investigated 6 months before the study.

**Table 1:** Clinical characteristics of patients included in in vitro assays.

	Limited cutaneous SSc	Late diffuse cutaneous SSc	Early diffuse cutaneous SSc
Number	12	11	13
N females (%)	10 (83)	9 (82)	11 (85)
Age at onset	42.6±12.3	40.6±11.2	44.3±10.2
Disease duration	9.1±7.8	7.9±7.1	1.1±0.7
ANA positivity	100%	73%	92%
mRSS at inclusion	not assessed	15.8±8.3*	22.2±8.5*
Pulmonary hypertension	33%	18%	15%
Lung fibrosis	25%	45%	31%
Current Therapies			
MMF	0%	36%	30%
Cyclophosphamide	0%	18%	15%
Prednisolone	25%	28%	53%
Hydroxychloroquine	17%	9%	0%
anti-IL-3	0%	0%	8%
Methotrexate	0%	0%	0%
Tacrolimus	8%	0%	0%

* 0.03.

#### Measurement of circulating cytokines

For the measurement of circulating levels of TNFα, IL-6 and IFNγ plasma from healthy controls (n = 28) and 177 SSc patients (lSSc n = 110, ldSSc n = 34, edSSc n = 33) from the Boston University Area (similar to those included in the *in vitro* studies), the Radboud University Nijmegen Medical Center area (RUMC) and Lund University Hospital area were analyzed. Blood and plasma samples were obtained with approval by written informed consent under Institutional Review Board approval protocols at all three academic centers involved.

### Monoclonal antibodies

For immunostaining and analysis by fluorescence-activated cell sorting (FACS), we used phycoerythrin (PE), allophycocyanin (APC) and fluorescein isothiocynate (FITC) conjugated mouse monoclonal antibodies (mAb) against human CD4, CD8, GITR, CD69, IL-23R, CD45Ro, CD45Ra, and CD25 (Miltenyi Biotec Inc., CA, USA). Intracellular staining of CD45Ro, CD45Ra or CD25+ cells for IL-17, TGFβ and IFNγ was performed using the intracellular staining procedure according to the manufacturer's protocols. Corresponding mouse/rat isotype controls were included in all analyses.

### Isolation of PBMCs, CD3^+^ cells and flow cytometry

PBMCs were isolated from heparinized venous blood by using density-gradient centrifugation over Ficoll-Paque (Amersham Bioscience). Next, CD3+ cells were isolated from PBMCs using CD3 microbeads according to manufacturer's protocol (Miltenyi Biotec). After isolation, cells were directly transferred into RPMI 1640 media supplemented with 2 nM L-glutamine, 100 U/µL/ml penicillin/streptomycin (Life technologies), and 10% FBS (BioWhitacker) in 96-well U-bottom plates (Nunc). For flowcytometric analysis, CD3+ were kept on ice and washed extensively with citrated PBS containing 1% FCS. 10 µl of FITC, APC or PE- conjugated antibody was added and incubated on ice for 20 min. 300 µl FACS buffer was added and T cells were pelleted, resuspended in 200 µl buffer. Thereafter, cells were washed in buffer, fixed with 2% formaldehyde, washed again in buffer and stored at 4°C. The cells were analyzed using a LSRII FACScan flow cytometer (BD Biosciences) and data were processed using FlowJo software.

#### Measurement of intracellular and circulating cytokines

Intracellular expression of IL-17, IFNγ and TGFβ in CD4 and/or CD25^high^ cells was investigated using monoclonal antibodies obtained from BD Bioscience, NJ, USA. After the staining protocol, cells were fixed with 2% formaldehyde, stored at 4°C and analyzed on flow cytometer the next day. Circulating and supernatant cytokine levels were measured and analyzed with the Bio-Plex system (Bio-Rad). TGFβ in supernatants was measured using the sensitive assay first described by Abe et al. [29] whereas IFNγ and IL-17 were measured using the Luminex platform. The sensitivity of the cytokine assay was <5 pg/ml for all cytokines measured.

#### Statistical analysis

Values are shown throughout the paper as mean±sem. Proportions of lymphocyte subpopulations were compared using the Student's t test for normally or not normally distributed population where appropriate. Relationships between different values were examined using Pearson's correlation coefficient and Spearman's rank correlation tests. Difference between groups were calculated using the Mann-Withney U test. All statistical analyses were performed using Graphpad Prism (GraphPad Prism 4.0 by Graph Pad software Inc.).

## Results

### CD4 positive T cells from SSc patients display an activated phenotype

**Figure 1:**
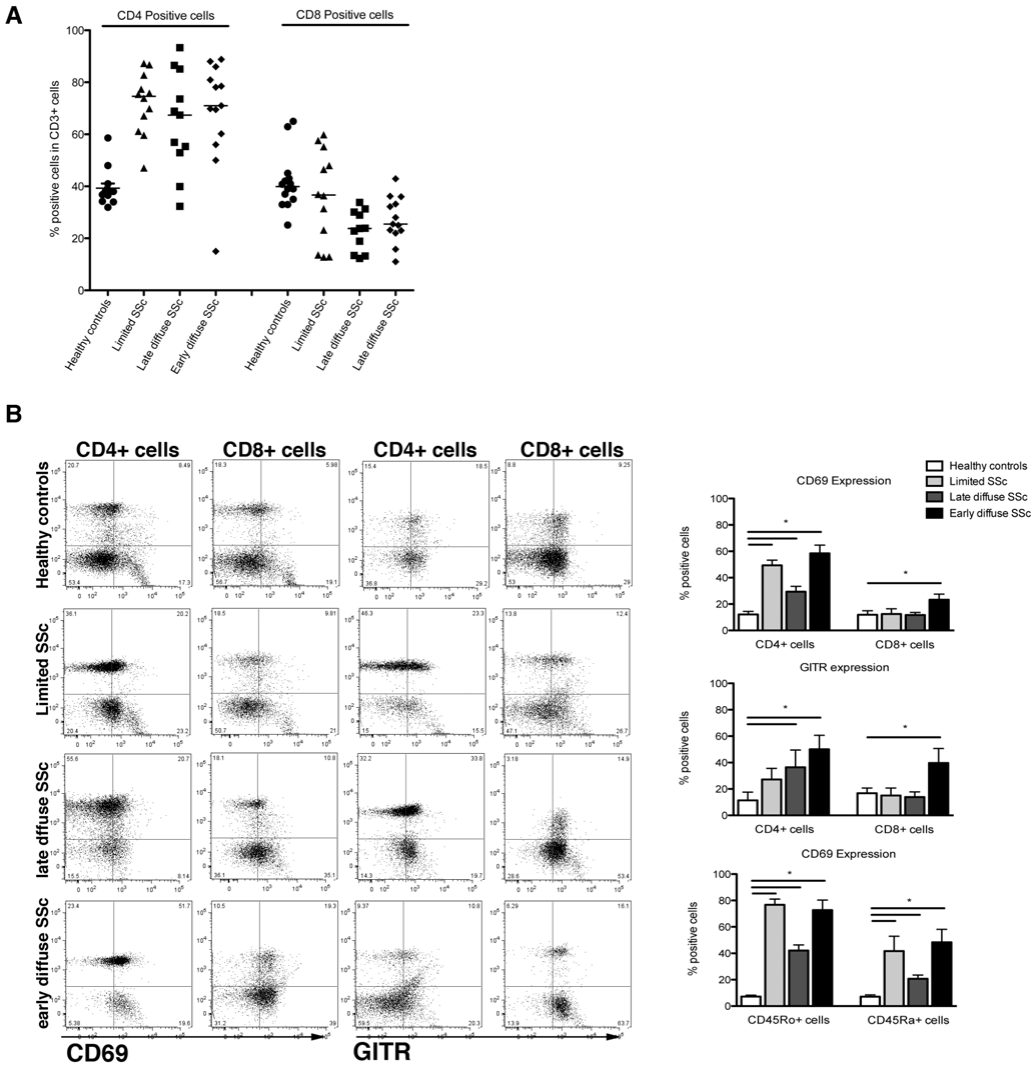
SSc patients have more and more activated CD4+ T cells compared to healthy controls. Panel A depicts the percentage of CD4+ and CD8+ cells in the whole T cell pool (CD3+ cells) from healthy controls (n = 14), and SSc patients with the lcSSc (n = 12), ldcSSc (n = 11) and edcSSc (n = 13) phenotype. The CD3+ cells were isolated using MACS bead isolation after which CD4 and CD8 positivity was analyzed using flow cytometry. Panel B (left) depicts the percentage of CD4+ and CD8+ cells that were double-positive for the T cell activation markers CD69 and GITR. For this aim a representative individual from each group was selected. In the right panel, the percentage of CD4-CD69, CD8-CD69, CD4-GITR, CD8-GITR, CD45Ro-CD69 and CD45Ra-CD69 double positive cells is presented over the whole group of healthy donors (n = 14) and/or SSc patients (n = 36).

Previous reports have described an increased CD4/CD8 ratio in SSc patients compared with healthy controls, however, recent markers permit a more refined analysis of T cell phenotype [2], [30]. Since effector T cells, suggested to be involved in SSc, arise from the CD4+ T cells, we first investigated T cell phenotype and activation. Consistent with previous studies, healthy controls (n = 13) displayed a considerably lower CD4+/CD8+ ratio than that observed in SSc patients (P<0.0001, **figure 1a** **)**. We next investigated whether CD4+ cells in SSc patients expressed T cell activation markers, CD69 and GITR. Indeed, SSc patients on average expressed significantly higher levels (*P*<0.0001) of CD69 on CD4+ effector T cells compared with those from healthy controls **(** **figure 1b** **)**. In contrast, CD69 expression was increased on CD8+ cells only from those patients with the edcSSc, but not on CD8+ cells from patients with lcSSc or ldcSSc (P<0.001). The expression of GITR displayed a similar pattern being higher on CD4+ cells from all subgroups of SSc patients compared to controls (P<0.001). Highest expression was seen in patients with edcSSc, with progressively less expression seen in patients with ldcSSc and lcSSc, respectively **(** **figure 1b** **, left panel)**. These observations prompted us to further study the expression of these activation markers on memory (CD45Ro) and naive T cells (CD45Ra), since CD45Ro cells were previously found to be the main producers of IL-17. Interestingly, the expression of CD69 was significantly higher on both CD45Ro and CD45Ra positive T cells in all SSc patients compared with healthy controls, suggesting that both these cell populations are activated in SSc **(** **figure 1b** **right panel)**.

### Th17 cells are more frequent in SSc patients and IL-17 in combination with IFNγ and TGFβ expression in T cells discriminates SSc subsets

**Figure 2:**
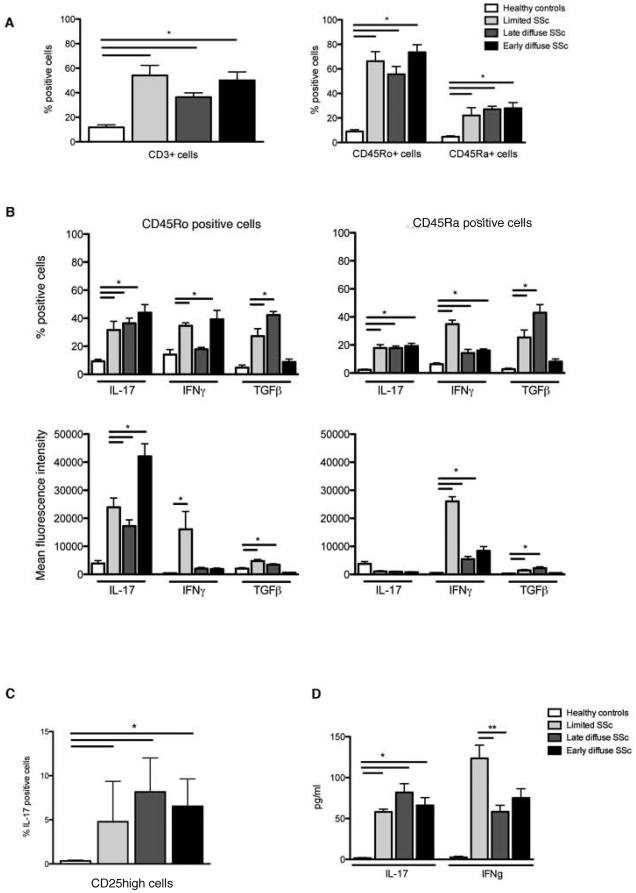
SSc patients express high levels of IL-17-positive T cells and the co-presence of IL-17 with either IFNγ, IFNα or TGFβ reflects SSc phenotype. Panel A presents the percentage of IL-23R positive cells in the CD4+ positive pool of T cells (left panel) and CD45Ro and CD45Ra positive cells (right panel) from healthy controls (n = 14), and SSc patients with the lcSSc (n = 12), ldcSSc (n = 11) and edcSSc (n = 13) phenotype. Panel B shows the mean intracellular expression level of IL-17, IFNγ and TGFβ in CD4+ cells from each a representative individual from each tested group (left side). On the right side, the mean percentage of CD45Ro-positive or CD45Ra-positive cells that express IL-17, IFNγ or TGFβ and the mean intensity thereof are presented for the whole group of healthy controls (n = 14), and SSc patients with the lcSSc (n = 12), ldcSSc (n = 11) and edcSSc (n = 13) phenotype. Panel C depicts the percentage of CD25^high^ cells that co-express IL-17 in healthy controls and different SSc phenotypes (* represents a p-value<0.01). Panel D represents the level of cytokines (IL-17 and IFNγ) spontaneously secreted by CD3+ T cells from healthy controls and SSc patients after 24 hrs of incubation. * *P*-value<0.0001, ** *P*-value<0.002, *** *P*-value<0.004.

These observations showing activated T cells in patients with SSc and recent findings of increased circulating levels of IL-17 and IL-23 in patients with SSc, led us to examine the expression of the IL-23 receptor (IL-23R) on T effector cells. Since it was recently demonstrated that IL-23 is pivotal in the survival of Th17 cells, increased expression of IL-23R might lead to enhanced Th17 cell survival in patients with SSc [8], [15], [31], [32]. Intriguingly, the expression of IL-23R was markedly higher on CD3+, CD45Ro+ and CD45Ra cells from all SSc patients investigated **(P<0.0001,** **figure 2a** **)**. Notably, increased IL23R expression was observed on CD4+ T cells of patients with both limited and diffuse cutaneous SSc, including patients with ldSSc. Next, we investigated the intracellular expression of IL-17 in CD45Ro and CD45Ra cells. Consistent with the increased expression of IL-23R and markers of activation (CD69 and GITR expression), the number of CD45Ro cells that co-expressed IL-17 was significantly increased in all SSc patients investigated **(P<0.0001,** **Figure 2b** **)**. As previously described, CD45Ra cells from healthy controls did not express IL-17. In contrast, CD45Ra cells from SSc patients showed increased numbers of IL-17 expressing cells that reached almost similar levels as CD45Ro cells However, the mean fluorescence intensity (MFI) of IL-17 in CD45Ro cells in SSc patients was clearly increased compared to that observed in CD45Ra cells.

To further investigate cytokine expression of T cell from SSc patients, we investigated the expression of TGFβ and IFNγ other cytokines that have been implicated in SSc pathogenesis. Comparison of the cytokine expression by T cells from patients with the different SSc phenotypes revealed a clearly distinct pattern. For instance, IFNγ was highly expressed by both CD45Ro and CD45Ra cells from patients with limited SSc, was almost absent in CD45Ro cells from SSc patients with diffuse SSc and expressed at intermediate levels by CD45Ra cells from diffuse patients. In contrast, the expression of TGFβ was increased in both CD45Ro and CD45Ra cells from patients with limited and late diffuse SSc but was normal in early diffuse patients. Based on these observations we propose that certain cytokine patterns are associated with certain SSc subtypes **(** **Table 2** **)**.

**Table 2:** Cytokine expression pattern in SSc clinical phenotypes.

Phenotype/Cytokine expression	IL-17	IFNγ	TGFβ
Healthy controls	low	Low	low
Limited SSc	high	High	high
Late diffuse SSc	high	low	high
Early diffuse SSc	high	intermediate	low

Previously, we demonstrated that SSc patients have increased levels of circulating T regulatory cells (submitted for publication). Recently, two elegant studies revealed that neither Tregs nor Th17 cells are terminally differentiated but, under pressure of several cytokines or other immune cells, could switch phenotype [33]. Since this could be an explanation for concurrent increased expression both of Tregs and Th17 in SSc, we investigated whether CD25^high^ expressing cells (Tregs) co-expressed IL-17. Indeed, significant higher numbers of CD25high/IL-17-positive cells were observed in SSc patients compared with healthy controls (P<0.01, **figure 2c**).

To further extend and confirm our results showing increased intracellular expression of IL-17 and IFNγ in SSc, we next measured the levels of IL-17 and IFNγ in the supernatant of CD3+ T cells isolated from healthy controls (n = 5), lcSSc patients (n = 5), ldcSSc patients (n = 5) and edcSSc patients (n = 5). Consistent with intracellular cytokine expression, T cells from all three clinical phenotypes secreted high levels of IL-17 and IFNγ compared to T cells from healthy controls (**figure 2d**). The pattern of altered secretion was also similar, with the highest levels of IFNγ secreted by T cells from patients with lcSSc, while the highest levels of IFNγ were secreted by T cells from patients with early diffuse SSc.

### SSc patients have normal levels of IL-17 but increased levels of Th17 promoting cytokines IL-1α, IL-23 and IL-6

**Figure 3:**
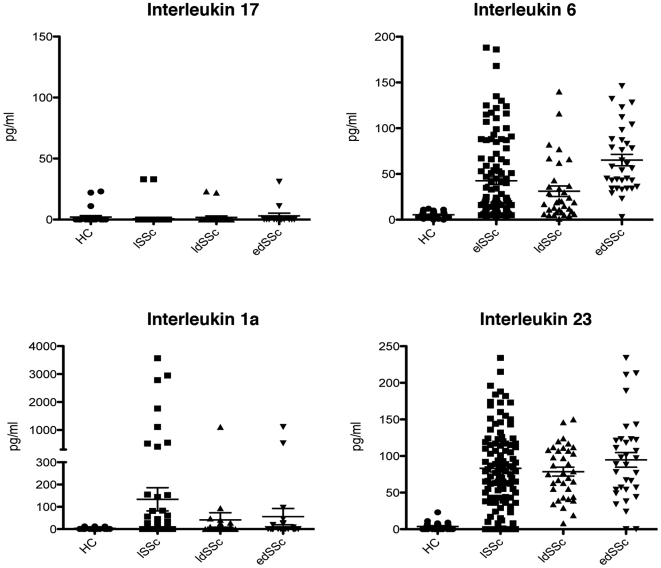
Increased level of Th17 inducing cytokines in the circulation of SSc patients. Panel A depicts the presence of IL-17 in the circulation of SSc patients and healthy controls. Panel B, C and D represents the levels of IL-6, IL-1α and IL-23, respectively. The circulating levels of IL-17, IL-1α and IL-23 was measured by ELISA whereas IL-6 was studied by Bioplex assays.

It has previously been reported that IL-17 levels are increased in the circulation in SSc patients [15]. A major limitation of these studies was the small sample size. This, together with our experience that circulating IL-17 is very difficult to detect in other autoimmune disorders such as rheumatoid arthritis, we investigated the levels of IL-17 and IL-17 promoting cytokines in a large cohort of SSc patients (n = 177) consisting out of 110 patients with LSSc, 34 with ldSSc and 33 with the edSSc phenotype. As a comparator group we used 28 healthy controls. When comparing the level of IL-17 in SSc patients and healthy controls, IL-17 could only be detected in a minority of the samples (9 of 177; **Table 3**, **Figure 3a**) and was not detected more frequently in SSc patients compared to controls. In contrast, the levels of IL-6 (53.6±9.7 vs. 5.4±3.4, P<0.0001), IL-1α (83.2±11.2 vs. 1.2±1.1, P<0.002) and IL-23 (49.1±7.3 vs. 5.3±0.6, P = 0.003) were significantly higher in SSc as a whole compared with controls **(** **Figure 3b–d** **)**. Subgroup analysis revealed that IL-6 and IL-23 levels were equally distributed among SSc phenotypes whereas IL-1α was significantly increased in lSSc patients (P<0.001) only. An association between clinical characteristics including the presence of autoantibodies, disease duration and/or pulmonary involvement was not observed (data not shown).

**Table 3:** Clinical characteristics of patients used for measurement of circulating cytokines.

	Limited cutaneous SSc	Late diffuse cutaneous SSc	Early diffuse cutaneous SSc
Number	110	34	33
N females (%)	89 (81)	26 (76)	27 (82)
Age at onset	43.3±8.9	42.7±11.7	41.3±9.8
Disease duration	11.2±7.1	8.1±5.3	1.5±0.8
ANA positivity	100%	77%	92%
Pulmonary hypertension	37%	19%	18%
Lung fibrosis	22%	47%	30%
Current Therapies			
MMF	0%	7%	8%
Cyclophosphamide	0%	16%	33%
Prednisolone	14%	11%	23%
Hydroxychloroquine	3%	0%	0%
anti-IL-3	0%	0%	0%
Methotrexate	0%	0%	0%
Tacrolimus	0%	0%	0%

## Discussion

We show here that patients with SSc have strikingly increased frequencies of circulating Th17 cells. In addition, the combined analysis of intracellular IL-17 with the expression of IFNγ or TGFβ revealed a pattern among patients that correlated with the different clinical SSc phenotypes. In line with these observations, levels of Th17 inducing cytokines IL-6, IL-1α and IL-23 were significantly higher in the circulation of SSc patients compared to controls, although circulating IL-17 was not detectable. Similar to our observations, the measurement of IL-17 in the circulation of patients with rheumatoid arthritis and psoriasis, two conditions in which IL-17 play a pivotal role in local pathology, was very disappointing. Therefore, it is tempting to speculate that IL-17 might be an important cytokine locally in SSc.

In our study all SSc patients had increased frequencies of IL-17 positive cells and T cells cultured from SSc patients showed high spontaneous production of IL-17. However, co-expression of IFNγ and TGFβ together with IL-17 distinguished SSc phenotypes. Patients with lcSSc expressed IL-17, IFNγ and TGFβ at the highest levels. In particular, T cells from these patients expressed higher levels of intracellular IFNγ than patients with edcSSc. Interestingly, in the lcSSc subgroup circulating levels of IL-1α were significantly higher compared to other SSc phenotypes and healthy controls, whereas IL-6 and IL-23 levels were elevated but comparable in all SSc subgroups. Perhaps, higher IL-1α levels skew the balance to more IL-17/IFNγ double positive T cells.

The potential involvement of IL-17 in various autoimmune diseases has sparked research aimed at the identification of the forces driving Th17 priming. To date, accumulating evidence point towards the essential role of DCs in orchestrating Th17 priming by the production of the driving factors for Th17 development, such as TGFβ, IL-1α, IL-6 and IL-23. More recently, it has become clear that Toll-like receptor mediated DC activation is also implicated. In this light Gerosa and co-workers demonstrated that the combination of specific Toll-like receptor (TLR) ligands dramatically stimulated IL-23 production and skews the immune response towards Th17 [34]. Although the role of TLRs in SSc has not been subjected to extensive research, our observations suggest a possible role for TLRs as a stimulus for the increased numbers of Th17 cells in these patients. TLR are critical for the innate immune response and bridge the innate and adaptive immune response [35]. Many ligands have been described for TLRs [36]. For TLR2 and TLR4 both exogenous (derived from microorganisms) and endogenous (originating from “self” tissues) have been identified. In contrast, ligands identified for the intracellular TLR3, TLR7, TLR8 and TLR9 mainly comprise exogenous ligands including double and single stranded RNA and CPG DNA. In this light it is interesting that several endogenous ligands for TLR4 are present in the plasma of some SSc patients ([37], unpublished results). We are currently investigating the nature of endogenous TLR ligands in different SSc phenotypes that could help to explain the observed differences with respect to co-expression of several cytokines in conjunction with IL-17. Recent observations from our group indicate an aberrant TLR responses in SSc that are distinct among patients having lcSSc, ldcSSc and edcSSc (manuscript submitted).

Little is known about the differentiation and maturation of IL-17 positive cells in humans. In contrast with the initial reports, we demonstrate that the Th17 phenotype is not confined to CD4+ effector cells but also includes a substantial number of naïve cells (CD45Ra) [11]. The latter is in line with a recent report investigating Th17 cells in seronegative spondylarthropathy [22]. As explained in this report, potential differences between studies could be explained by slightly differences in isolation protocols. However, an important other explanation might be that the factors that drive Th17 among different diseases differ also with respect to the CD4+ subpopulations that are activated.

Taken together, although the underlying mechanisms that explain the distinct patterns of intracellular cytokine expression among SSc phenotypes need to be identified, these patterns suggest distinct immune dysregulation in dcSSc versus lcSSc and in early versus late disease in dcSSc. These insights open novel avenues for research aimed at identifying pathogenic pathways and therapeutic targets.
